# The Use of Medicinal Plants for the Treatment of Toothache in Ethiopia

**DOI:** 10.1155/2019/2645174

**Published:** 2019-08-20

**Authors:** Moa Megersa, Tilahun Tolossa Jima, Kabaye Kumela Goro

**Affiliations:** ^1^Department of Biology, Maddawalabu University, P.O. Box 247, Robe, Ethiopia; ^2^Department of Biology, Ambo University, P.O. Box 19, Ambo, Ethiopia; ^3^Department of Clinical Pharmacy, Jimma University, P.O. Box 378, Jimma, Ethiopia

## Abstract

This paper presents a review of relevant medicinal plants used for toothache treatment in Ethiopia. This finding is based on a review of the literature published in scientific journals. A total of 130 medicinal plants, distributed in 117 genera and 62 families, are reported in the reviewed literature. Of the 130 species of medicinal plants reported in the literature, ninety-two (70.7%) were obtained from the wild whereas twelve (9.2%) were from home gardens. Shrubs (34.6%) were the primary source of medicinal plants, followed by herbs (30%). The Asteraceae came out as a leading family with 12 medicinal species while the Fabaceae followed with nine. Some findings include the predominance of root material used (31%), followed by leaves (29%). This study demonstrates the importance of traditional medicines in the treatment of toothache in Ethiopia. It is essential for the health of users to phytochemically demonstrate the effects of medicinal plants for their possible therapeutic applications. Hence, future phytochemical and pharmaceutical studies should give due consideration on frequently reported medicinal plants in order to produce natural drugs that could be effective in toothache treatment and without side effects.

## 1. Introduction

### 1.1. Toothache

Toothache is a common problem occurring in the human population throughout the world frequently. The World Health Organization (WHO) recommended the reduction of toothache as one of the priority issues in the global oral health promotion agenda [1]. Toothache is defined as an orofacial pain originated from a dental element and/or adjacent structures in consequence of several diseases or conditions, such as dental caries, periodontal disease, trauma, occlusal dysfunction, and abscess [2]. The causative factors behind toothache include tooth decay or fracture, abscessed tooth, or infected gums [3]. Over 750 species of bacteria inhabit the oral cavity and a number of these are implicated in oral diseases including toothache [4]. The development of dental caries involves acidogenic and aciduric Gram-positive bacteria, primarily the *Streptococcus* species, *Lactobacillus*, and *Actinomycetes*, which metabolize sucrose to organic acids that dissolve the calcium phosphate in teeth [5, 6].

Toothache is prevalent in lower socioeconomic status groups and in populations where dental caries is largely untreated [7–9]. It affects the sleep, feeding, work performance, and productivity [10]; if not treated well can lead to the loss of tooth [3]. In children, the pain can affect school attendance, eating, and speaking and then impair growth and development [11, 12]. The prevalence of dental caries in school-aged children is up to 90% in many parts of the world where the adults are also affected [12]. Epidemiological studies on toothaches conducted elsewhere in Ethiopia indicated that toothache mainly due to dental caries is prevalent in school-aged children. For instance, a study conducted in Finote Selam showed that 48.5% of the students had dental caries [13]. A similar study by Tafere et al. [14] reported that dental caries was 72.8% prevalent among study groups in Debre Tabor, Ethiopia. This indicates the need for improved diagnostic and therapeutic procedures in dentistry, especially in children [15]. However, access to dental healthcare is limited in most developing countries including Ethiopia and is generally restricted to emergency dental care or pain relief [12]. Thus, visiting dentists is unaffordable and many local communities treat a toothache at home mainly of using plant species as a chewing stick [16].

### 1.2. Medicinal Plants for Toothache Treatment

Medicinal plants have been used as traditional treatments for numerous human diseases for thousands of years and in many parts of the world [6]. According to the World Health Organization, between 65% and 80% of the populations of developing countries use medicinal plants as remedies [17] and the use of traditional medicine continues to expand rapidly across the world [18]. In Africa, the dependence on traditional medicine is linked with poverty, the inadequacy of health services, and a shortage of drugs [19, 20].

The use of medicinal plants has a long history in dental practice, and they have long been used worldwide [6]. There have been numerous reports of the use of traditional plants and natural products for the treatment of toothaches. For instance, the result of a study in Tanzania indicated that dental patients are commonly treated by traditional healers using medicinal plants [21]. In Cameroon, 32 medicinal plants are used in the treatment of toothache [20]. Local communities in Burkina Faso used 62 medicinal plant species for the treatment of oral diseases, of which 41 plants are utilized for the treatment of toothache alone [22]. In Madagascar, local communities of Mahajanga used 63 plant species to treat dental caries and 23 plants to treat periodontal diseases [23]. A similar study conducted by Ngari et al. [24] and Delfan et al. [25] also showed that local people in Kenya and Lorestan Province of Iran used 12 and 14 medicinal plants, respectively, in order to get relief from toothache.

Like other countries, local communities in Ethiopia use medicinal plants to treat a toothache at a household level to get relief from the disease. *Acmella caulirhiza* [26, 27], *Allium sativum* [28, 29], *Datura stramonium* [30], *Clausena anisata* [28], and *Solanum incanum* [30] are among the plant species frequently used by local people in Ethiopia, out of which *Datura stramonium* appeared to be the frequently used plant for toothache treatment.

Herbal extracts have been used in dentistry for reducing inflammation, for inhibiting the growth of oral pathogens, for preventing the release of histamine, and as antiseptics, antioxidants, and analgesics [6, 18]. Various phytochemical studies conducted on medicinal plants traditionally used for toothaches proved the presence of active compounds against oral pathogens. However, many studies investigating the activity of traditional medicinal plants against oral pathogens have been limited to the examination of crude extracts [6, 18]. For example, the methanol extracts of aerial parts of *D. stramonium* showed the bactericidal activity against Gram-positive bacteria, whereas the ethanol extract exhibited the highest inhibitory against *Staphylococcus aureus* which is an oral bacterium [31]. A similar study conducted by Balto et al. [32] on the effectiveness of *Salvadora persica* which is a toothbrush tree traditionally used for oral hygiene in Ethiopia [33] showed an inhibitory effect on oral bacteria. They used ethanol and hexane to extract active compounds from the plant species. Moreover, studies have shown that alcoholic solvents have more antimicrobial activity than aqueous *S. persica* extracts [32]. In the study of purified phytochemicals against oral pathogens, flavonoids, alkaloids, terpenes, and others showed an inhibitory effect against oral bacteria [6]. For example, tropane alkaloids, atropine, and scopolamine were isolated from *D. stramonium* [34, 35], two active isoprenylflavones, artocarpin and artocarpesin, were isolated from *Artocarpus heterophyllus* [36], and phytochemical screening of *Clausena anisata* revealed the presence of tannins, alkaloids, steroids, saponins, phenolics, and flavonoids [37]. These purified phytochemicals inhibited the growth of numerous oral bacteria responsible for toothache [6].

This review describes the traditional uses of medicinal plants used for toothache treatment in Ethiopia. We also reviewed the experimental evidence that has served to confirm the traditional use of medicinal plants to inhibit the growth of oral pathogens responsible for toothache. Moreover, this review is initiated to identify research gaps and to suggest perspectives for future research in the development of drugs.

## 2. Methods

The traditional uses of medicinal plants used to treat toothache in Ethiopia were collected from available literature published in scientific journals, books, theses, proceedings, and reports. Literature was searched in PubMed, PMC, Science Direct, and Google Scholar databases and accessed between April 2018 and January 2019 using specific search terms such as “medicinal plants,” “traditional medicines,” and “Ethiopia or Indigenous people”. After identifying potential literature, we searched if there is a report of medicinal plants used for toothache, tooth decay, tooth problems, tooth infection, and tooth pain in the region where the study was carried out. Hence, papers that do not report the use of plant species for toothache treatment were omitted. In addition, studies that reported the use of plant species for brushing purpose were excluded. However, studies that reported the plant species used as brushing for the toothache treatment were included. Data collected from the literature include demography of respondents, year of publication, habit, habitat of the species, preparation methods, plant parts used, and condition. Moreover, a literature search was also conducted to document the biological and pharmacological activities of frequently reported plant species for toothache treatment such as *D. stramonium*, *Olea europaea*, *A. caulirhiza*, and *S. incanum*. The plant names were directly extracted from the literature and validated using the website (http://www.theplantlist.org).

We reviewed a total of 179 ethnobotanical studies conducted in Ethiopia. A total of 72 studies met the criteria (reporting treatment of anti-toothache/tooth problem using plant species) and were included in the review. The publications reported the use of medicinal plant species to treat toothache in Ethiopia. A list is produced, showing scientific names, parts used, habit, and references for each species (Table 1).

## 3. How Many Studies Were Reported on the Use of Medicinal Plants for Toothache Treatment?

**Figure 1:**
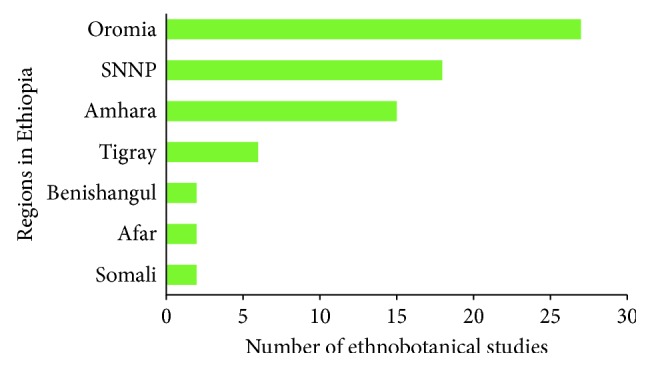
Number of ethnobotanical studies in Ethiopia that reported the use of medicinal plants for toothache treatment.

**Figure 2:**
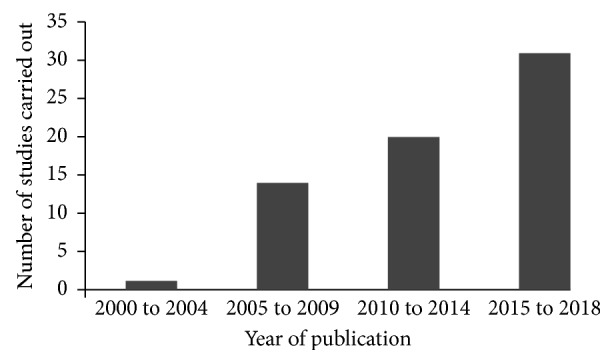
Number of ethnobotanical studies (toothache) in Ethiopia per year.

A total of 72 ethnobotanical studies performed in Ethiopia that reported the use of medicinal plants for the treatment of toothache were identified (Figure 1). The 72 studies generally reported the use of plant species for human health treatment including toothache. However, no research was conducted specifically on toothache treatment. Of the studies, 27 (37.5%) were carried out in the Oromia region, 18 (25%) in SNNP (South Nation and Nationalities Peoples) region, 15 (21%) in Amhara, and 6 (8.3%) in Tigray region (Figure 2). A review by Alebie et al. [102] on antimalarial plants and Woldeab et al. [103] on antidiarrheal plants in Ethiopia indicated a similar result as many studies were conducted in Amhara, Oromia, and SNNP regions. However, Benishangul, Afar, and Somali regions have received less attention so far; hence, studies should be conducted in these regions as the ethnomedicinal knowledge varies even in the same ethnic group.

The published ethnobotanical studies in Ethiopia are also increasing from year to year. For example, we found one article [39] reporting the use of plants for toothache treatment between 2000 and 2004 and the number increased to 31 between 2015 and 2018 (Figure 2). In agreement, Albuquerque et al. [104] highlighted that ethnobotanical studies are increasing in Brazil which could demonstrate the remarkable growth of ethnobotany as a science.

### 3.1. Taxonomic Diversity of Medicinal Plants Used for Toothache Treatment in Ethiopia

We report on a total of 130 medicinal plant taxa, belonging to 112 genera and 62 families used by Ethiopian people for the treatment of toothache (Table 2). Among the families that contributed more medicinal species were the Asteraceae, represented by 12 species (9.2%), Fabaceae by 9 (7%) species, and Solanaceae by 5 (4%) species, and other 59 families contributing 104 (80%) species are represented by 1 to 4 species (Table 2). The finding of the family Asteraceae as the contributor of the higher number of plant species used for toothache treatment than other families agrees with a review study conducted on anticancer plants in Ethiopia [105]. A review by Uprety et al. [106] and Kumar [107] also indicated that local communities in the boreal forest of Canada and India prepare remedies for oral health and other disease treatment mainly from Asteraceae family. On the other hand, other researchers reported that Fabaceae is the leading family with the highest number of medicinal plants in various diseases treatment in Ethiopia [102, 103] or elsewhere in the world [108, 109]. Both findings are reasonable since the two families are both represented by a higher number of species in Ethiopian flora [48]. Of the 130 species of medicinal plants reported from the literature, most of them (92, 71%) were obtained from the wild whereas 26 (20%) were from both home gardens and wild habitats, and only 12 (9%) species were from home gardens.

**Figure 3:**
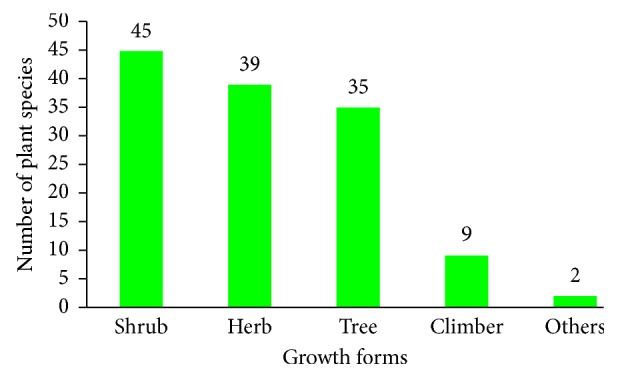
Growth forms of medicinal plants used in the treatment of toothache in Ethiopia.

The result of the growth from analysis of medicinal plants used for toothache treatment in Ethiopia showed that shrubs constituted the highest proportion being represented by 45 (34.6%) species, while there were 39 (30%) herb species and 35 (27%) trees (Figure 3). The dominance of shrubs for remedy preparation for toothache treatment is in line with a review by Alebie et al. [102] and Esubalew et al. [105] on anticancer and antimalarial activity of plant species in Ethiopia. The dominance of shrub for toothache treatment is reasonable as many medicinal plants are being used as a toothbrush. Moreover, it was reported that the availability of shrub plant species throughout the year due to their relative capability of resisting drought and seasonal variation could aid in extensive uses of shrub species compared to herbaceous plants [110].

### 3.2. Plant Parts Used in Toothache Treatment

**Figure 4:**
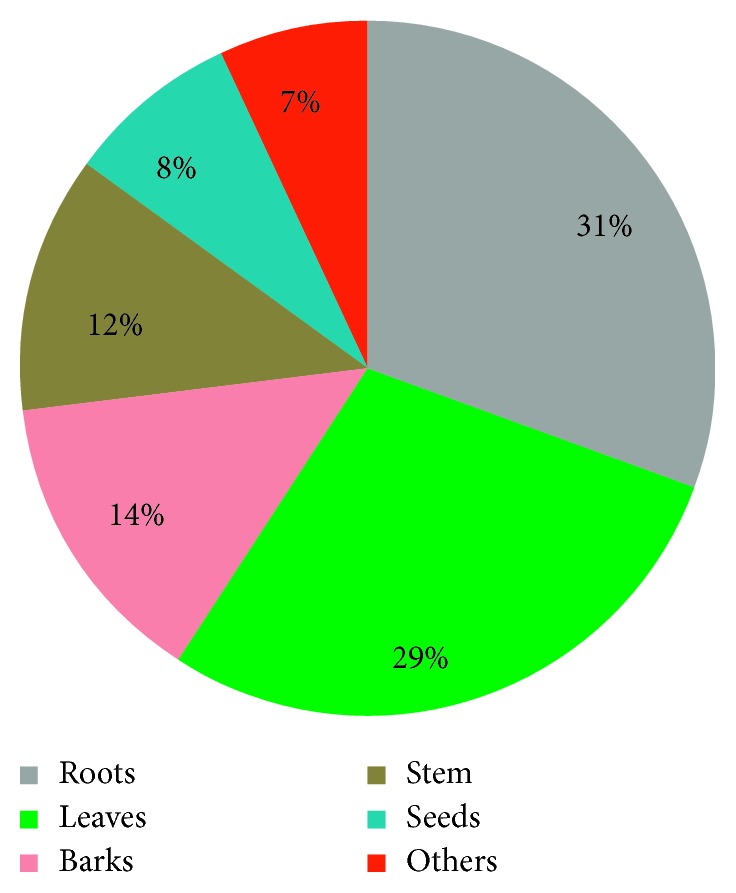
Plant parts used for the treatment of toothache in Ethiopia.

Local people of Ethiopia harvest different plant parts for preparation of traditional drugs for toothache treatment (e.g., leaves, roots, seeds, barks, and fruit). In Ethiopia, various authors reported that about 31% of medicinal plants were harvested for their roots and these were followed by leaves (29%) and barks (14%) (Figure 4). The utilization of roots for drug preparation is not a good practice as it threatens the survival of the plant species. Moreover, studies are indicating that overcollection of root parts for remedy preparation poses a threat to medicinal plants as it was observed in many plant species where the roots are utilized [26, 48].

### 3.3. Condition and Preparation of Traditional Medicine for Toothache Treatment

Most of the remedies (85%) in Ethiopia used for toothache treatment are prepared from fresh parts of medicinal plants followed by dried form 9% and 6% prepared either from dry or fresh plant parts. Most of the medicinal plant's preparations involved the use of single plant species or a single plant part (97%) while those mixing different plants or plant parts (3%) were rarely reported in the literature.

People living in Ethiopia use different traditional therapeutic methods to get relief from a toothache, which depends on the type of plant species. The plant extract, chewing, concoction, pounding, and decoction are the most common methods to treat the toothache. The most common methods of traditional medicine from plant material was chewing (56.5%), followed by decoction (9.7%), crushing (5.4%), and powdering and others (pounding, holding, rubbing, and inhaling) accounted 3.2% and 47%, respectively.

### 3.4. The Authors Consensus on Medicinal Plants Used to Treat Toothache in Ethiopia

Of 130 medicinal plants used to treat toothache, all species were not reported equally. Some medicinal plants were reported by various researchers as there are also a single species reported by a single author. For instance, 16 authors reported the use of *Datura stramonium* for toothache treatment followed by *Olea europaea* reported by nine authors, whereas 5 studies reported the use of *Acmella caulirhiza*, *Capparis tomentosa*, *Clausena anisata*, and *Premna schimperi* for toothache treatment in different parts of Ethiopia. The other six species (*Allium sativum*, *Ehretia cymosa*, *Euclea racemosa*, and *Solanum incanum*) were reported by four authors to be used in Ethiopian folk medicine to get relief from toothache. A review by Woldeab et al. [103] on antidiarrheal plants indicated that *Amaranthus caudatus*, *Calpurnia aurea*, *Coffea arabica*, *Cordia africana*, *Rumex nepalensis*, *Verbena officinalis*, *Verbascum sinaiticum*, *Vernonia amygdalina*, and *Zehneria scabra* are frequently reported plant species. To prioritize phytochemical and pharmacological studies on medicinal plants and to conserve the plants used for toothache treatment, this review could be used as baseline information.

### 3.5. Phytochemical Studies

Due to the increasing resistance of pathogens to conventional antimicrobial drugs, plant compounds are of interest as antiseptics and alternative antimicrobial substances [111]. To fully understand the pharmacological properties of medicinal plants, it is important to study phytochemistry of such plants [112]. Studies indicated that phytochemical insights into several plants that were similarly used in different countries have led to the isolation of novel structures for the manufacture of new drugs [113]. However, such studies are lacking in Ethiopia considering the vast number of plants used in traditional medicine for toothache and other disease treatment [103]. In recent years, phytochemical studies have been carried out to investigate medicinal plants used for toothache treatment.

A phytochemical study by Geyid et al. [113] has highlighted medicinal plants used to treat human diseases in Ethiopia. Among plants studied which showed inhibitory effect on oral pathogens were *Acacia nilotica*, *Albizia gummifera*, *Artemisia abyssinica*, *Clausena anisata*, *Clematis simensis*, *Cordia africana*, *Dovyalis abyssinica*, *Euclea divinorum*, *Jasminum abyssinicum*, *Momordica foetida*, *Pentas lanceolata*, *Stephania abyssinica*, *Verbascum sinaiticum*, and *Ximenia americana*. The authors also indicated that the species possess one or more of compounds among alkaloids, cardiac glycosides, polyphenols, tannins, unsaturated sterol, saponins, and glycosides. The phytochemistry of medicinal plants such as *Acmella caulirhiza* [114], *Allium sativum* [115], *Capparis tomentosa* [116], *Azadirachta indica* [117], *Datura stramonium* [34, 118, 119], *Ehretia cymosa* [120], *Euclea racemosa* [121], *Olea europaea* [122, 123], *Premna schimperi* [124], and *Solanum incanum* [125] has also been reported. For instance, the major phytochemicals isolated from *D. stramonium* are tropane alkaloids, atropine, and scopolamine [35]. Different alkaloids from seeds of *D. stramonium* were reported by Li et al. [126]. Sixty-four tropane alkaloids have been isolated from *D. stramonium* [119]. These alkaloids include *N*-trans-feruloyl tryptamine, hyoscyamilactol, scopoletin, umckalin, daturaolone, daturadiol, *N*-trans-ferulicacyltyramine, cleomiscosin A, fraxetin, 1-acetyl-7-hydroxbeta-carboline, and 7-hydroxy-beta-carboline-propionic acid. In addition, the phytochemical analysis of the plant revealed that *D. stramonium* contained saponins, tannins, and glycosides [118, 119].

Studies on chemical analysis of *A. caulirhiza* indicated the presence of lipophilic alkylamides or alkamides bearing a different number of unsaturated hydrocarbons such as spilanthol [114, 127] and amide derivatives [128]. Due to the presence of spilanthol, the plant possesses analgesic effect and induces saliva secretion [129–131]. In addition, phytosterols, essential oils, sesquiterpenes, *α*- and *β*-bisabolenes and cadinenes, flavonoid glucoside, and a mixture of long-chain hydrocarbons were reported [132, 133]. The phytochemical analysis of *A*. *sativum* confirmed the presence of allicin [134, 135]. In addition, the aqueous and methanolic extract of *A*. *sativum* indicates the presence of a rich number of secondary metabolites such as alkaloids, flavonoids, glycosides, cardiac glycosides tannin, phenolic compounds, saponins, terpenoids, and steroids [136, 137].

### 3.6. Pharmacological Studies

While the phytochemistry of many medicinal plants has been analysed, some Ethiopian plants still lack comprehensive scientific data to validate the pharmacological effects of their respective chemical constituents to treat toothache. Among the studies on the pharmacological effect of medicinal plants used for the toothache treatment include the effect of allicin extracted from *A. sativum*. The plant inhibits the growth of *Streptococcus mutans* and reduces its acid production. It also increases the secretion of saliva and can be effective for the prevention and treatment of dental caries [134, 135]. The extract also showed inhibition against *Porphyromonas gingivalis* [115]

Prashant et al. [138] and Hotwani et al. [139] examined the anti-toothache effect of *A. indica* and indicated that the extract reduces the frequency of early caries and reverses its process by decreasing the count of *S. mutans*, *S. mitis*, *S. sanguinis*, and *S. salivarius*. Pai et al. [140] also examined the pharmacological effect of the plant (*A. indica*) used to treat toothache and showed that extracts significantly decreased the plaque index and bacterial count.

Crude methanol, acetone, and chloroform extracts of *D. stramonium* exhibited antimicrobial properties against *S. mutans* and *Candida albicans* with varying inhibitory performances [141]. The minimum inhibitory concentration (MIC) reported by the authors was 80 mg/mL and 40 mg/mL against *S. mutans* and *C. albicans*, respectively. In a similar study conducted by Al-Ghamdi [142], the crude methanol leaves extract of *D.* stramonium showed no inhibitory activity against *S. mutans*, while the crude acetone extracts showed inhibitory activity at 4 mg/mL against *S. mutans*.

*O. europaea* (Oleaceae) is commonly known as olive tree. It is a tree bearing silvery green leaves and small white, feathery flowers [143]. *O. europaea* reported being an effective antimicrobial agent [144]. Stem extracts of *O. europaea* using petroleum ether, acetone, methanol, and water in soxhlet successively showed a broad spectrum of activity against microorganisms responsible for the most dental diseases [143]. Various authors reported that methanol extracts of *O. europaea* showed the maximum activity against *S. mutans* (16.6 mm) and *C. albicans* (13.6 mm). In another study by Sudjana et al. [145], the leaf extract showed activity against specific microbe and is not a broad-spectrum antimicrobial agent. Phenolic compounds from leaves of *O. europaea* also showed activity against *C. albicans* at low concentrations [146].

Bonou et al. [147] examined the activity of *C. anisata* on various oral pathogens and indicated that the extract from the plant is effective against *C. albicans* at 0.125 *μ*g/mL. In a similar study, dichloromethane and methanol extracts of *C. anisata* showed sensitivity at 8 mg·mL^−1^, 4 mg·mL^−1^, and 8 mg·mL^−1^ against *S. mutans*, *C. albicans*, and *Lactobacillus acidophilus*, respectively [148]. However, Kemoli et al. [149] observed no activity against *S. mutans* using the disc diffusion assay.

*S*. *incanum* fruits are locally used in Ethiopia to manage tooth decay, which is caused by mouth microbes [30, 49, 67, 68]. The pharmacological studies also proved that the fruit extracts of *S. incanum* inhibit the growth of oral microbes [150]. At the optimum concentration (70 *μ*l), oral microbes were inhibited (1.8). The authors also reported that alkaloids and solasodine found in fruits are responsible for antimicrobial activity.

### 3.7. Future Research and Viewpoints

This study showed that local people in Ethiopia rely on traditional medicines to treat toothache and are knowledgeable about the applications of medicinal plants. However, the dose and part used vary among place to place even in a specific plant species. For instance, different parts of *C. tomentosa* were reported to be used for toothache treatment. Wondimu et al. [40] and Kassa et al. [49] indicated that local people in Arsi and Ejere used roots and barks of this plant to get relief from toothache, respectively. In another study, Beyi [30] reported that the leaves of *C. tomentosa* are used in toothache treatment by local communities of Dugda district. These types of findings could show the urgency of phytochemical and pharmacological studies in order to prove or disprove its potency against oral microbes. In doing so, the most potent plant part will be investigated and applied in toothache treatment.

The current review addresses the existence of traditional indigenous knowledge in Ethiopia on toothache treatment. It is, therefore, necessary to preserve this indigenous knowledge on traditional medicines by proper documentation, identification of plant species or parts used, herbal preparation, and dosage [103]. This review will assist future studies on the selection of herbal plants used to treat toothache or oral pathogens in phytochemical and pharmacological evaluation. As a contribution to the ongoing search for alternatives, available, safe, and effective treatment to conventional drugs used to treat toothache, it is necessary to advocate scientific research on anti-toothache plants. Plant species which are being frequently utilized by different groups of people either in Ethiopia or in the world could be evidence for the activity of plant species on toothache treatment. For example, *D. stramonium* has been cited 16 times by different ethnobotanical studies conducted in different parts of Ethiopia [41, 44]. The pest prepared from this plant is also used for toothache treatment by local communities living in the central Himalaya of India [151]. Other ethnobotanical studies on oral health treatment also correlated the use of *S. incanum* for toothache treatment similar to Ethiopian communities. For example, local communities in Madagascar use the fruits of *S. incanum* through buccal inhalation for toothache treatment [23]. In a similar study, *C. tomentosa* which is a frequently utilized toothache plant in Ethiopia [30, 40, 49, 68] is also reported to be used as anti-toothache by local communities of Burkina Faso [22].

Although societies in Ethiopia have long used these plant species for toothache treatment with no health complaints, it is a good practice to perform toxicological tests before implementing the pharmacological results in a community. It needs a thorough scientific investigation mainly on toxicity aspects. For instance, a toxicological study on *D. stramonium* indicated that the plant is toxic when consumed improperly [31, 152] and the administrations of large amounts affect the central nervous system [31]. To offset the effect of dose and toxicity, attempts should be made to standardize the dose and authenticate plant species with anti-toothache properties [153].

Regarding the effectiveness of medicinal plants on the toothache, continuous studies should be done to confirm the local medicinal plant knowledge with a scientific approach. In different pharmacological studies, it was noted that crude extracts of the plant species were tested on oral pathogens [141–143]. However, purification of the active component is essential to elucidate the mechanism and aid in future drug development. It is also wise to study whether the components in crude extracts have a synergistic or antagonistic impact on oral pathogens inhibitory activity. The synergistic and antagonistic effect could be evaluated in both crude and fractionation (purified) form. If we decide to use the anti-toothache plants in their crude form, there might be a chance of achieving a synergistic effect and obtaining a better result. Studies have reported that pure drugs that are industrially produced or isolated from plants rarely have the same degree of activity as the unrefined extract at comparable concentrations or doses of the active component [154, 155]. This phenomenon is attributed to the absence of interacting substances present in the extract [154]. The synergy between different constituents of extracts has been documented in various pharmacological studies [155, 156].

A review by Woldeab et al. [103] highlighted that the number of informants who participated in ethnobotanical studies in Ethiopia is minimal similar to the finding of the current study. In this review, we found that the minimum number of participants was 30 in the study conducted by Birhanu and Ayalew [72] whereas the highest informants (1214) participated in the study by Flatie et al. [26]. The number of participants selected for ethnobotanical study in Ethiopia has no ground; rather, it depends on the will of researchers. In the future, a standard should be set on the number of informants to participate in ethnobotanical studies considering the geographical location, population size, and land size unless the sample size could not be representative to elucidate the medicinal plant knowledge of a given district [103]. Another concern of ethnobotanical studies conducted in Ethiopia is the age and sex of participants. Studies are concluding that the knowledge on medicinal plants is getting lost due to the lack of interest by the younger generation without concrete evidence [33, 98]. No comparative studies on the knowledge of medicinal plants have been made between young and old generations to reach the conclusion. In addition, a number of female participants were lower compared to male participants. On average, about 25 female respondents participated in each ethnobotanical study conducted in Ethiopia, whereas 64 males participated in each study. However, there were studies that collected data from an equal or a greater number of female participants [42, 55, 67, 85, 95]. Thus, future studies should focus on identifying gender-based knowledge differences related to medicinal plants use [103].

In Ethiopia, the knowledge of medicinal plants for toothache treatment only is poorly documented. Thus, future ethnobotanical studies should focus on the specific condition to gather as many as information related to the diseases. In doing so, a detailed preparation method, method of application, and other necessary information will be collected to aid future drug development.

## 4. Conclusions

The present study records 130 reported medicinal plants commonly used for toothache treatment in Ethiopia. The majority of traditional medicinal plants were harvested mostly from wild. In the study area, shrubs constituted the highest proportion of medicinal plants to be utilized for toothache treatment. Both leaves and roots are almost equally harvested to prepare the drug to get relief from the disease. The utilization of leaves may not cause a detrimental effect on the plants compared with plant species in which root is utilized. The review also found that medicinal plants such as *Acmella caulirhiza*, *Allium sativum*, *Capparis tomentosa*, *Clausena anisata*, *Datura stramonium*, *Ehretia cymosa*, *Euclea racemosa*, *Premna schimperi*, and *Solanum incanum* were reported by more than four researches in different parts of Ethiopia which might indicate the availability and efficacy of the plant species for toothache treatment. Hence, they have the potential to be developed into agents that can be used as a treatment therapy for toothache treatment. Study on the toxicological effects of plants should not be overlooked, as the main aim for studying indigenous plants is linked with searching safer and effective alternatives to modern drugs used for toothache treatment which are costly and very often require prolonged treatment.

## Figures and Tables

**Table 1:** Medicinal plants used for the treatment of toothache in Ethiopia. Description of languages.

Family	Scientific name	Local name	Growth habit	Part used	Preparation	References
Acanthaceae	*Barleria homoiotricha* C. B. Clarke		Shrub	Barks	Drink	[38]
	*Dyschoriste radicans* (Hochst. ex. Rich.) Nees		Climber	Whole		[39]
	*Justicia schimperiana* (Hochst. ex Nees) T. Anderson	Dhummuga (Or)	Shrub	Twigs	Chewed	[40]

Alliaceae	*Allium sativum* L.	Q/adii (Or)	Herb	Bulb	Crushed	[28]
				Bulb	Chewed	[29]
		Shingurti (Ti)		Bulb	Chewed	[41]
				Bulb	Chewed	[42]

Amaranthaceae	*Amaranthus caudatus* L.	Hamliadgi (Ti)	Herb	Roots	Chewed	[29]
		Chele Shullo (Ke)		Seeds		[43]

Anacardiaceae	*Rhus natalensis* Bernh. ex C. Krauss	Kubri (Ma)	Shrub	Leaves	Chewed	[44]
	*Schinus molle* L.	Q/barbare (Am)	Tree	Stem	Brushing	[45]

Apiaceae	*Foeniculum vulgare* Mill.	Arake (Am)	Herb	Roots	Decoction	[46]
	*Oenanthe palustris* (Chiov.) C. Norman	Itsesiol (Am)		Leaves	Chewed	[47]

Apocynaceae	*Calotropis procera* (Ait.) Dryand.		Shrub	Barks	Pounded	[38]
	*Carissa spinarum* L.	Agamsa (Or)	Shrub	Barks	Chewed	[48]

Araliaceae	*Schefflera abyssinica* (Hochst. ex A. Rich.) Harms	Arfaasee (Or)	Tree	Barks	Chewed	[49]

Asclepiadaceae	*Gomphocarpus purpurascens* A. Rich.	Tseba Dimu (Ti)	Herb	Roots	Chewed	[41]

Asparagaceae	*Asparagus africanus* Lam.	Yst kest (Am)	Shrub	Roots	Drink	[50]
		Serity (Or)		Roots	Chewed	[51]

Asteraceae	*Acmella caulirhiza* Del.	Etsegne (Br)	Herb	Root	Grounded	[26]
				Flowers	Chewed	[27]
		Yemidir Berbere (Am)		Flowers	Chewed	[44]
				Flowers		[52]
				Flowers	Chewed	[50]
	*Artemisia abyssinica* Sch.Bip. ex A. Rich.		Shrub	Stem	Chewed	[53]
	*Artemisia afra* Jack. ex Wild.	Ae'macho (Ke)	Herb	Leaves	Chewed	[43]
	*Echinops kebericho* Mesfin	Kebericho (Am, Or)	Herb	Root	Powdering	[45]
				Root	Pounded	[54]
	*Echinops macrochaetus* Fresen.	Qore harree (Or)	Herb	Root	Holding	[55]
	*Galinsoga parviflora* Cav.	Midirberber (Am)	Herb	Flower	Rubbing	[56]
	*Inula confertiflora* A. Rich.	Weinagift (Am)	Shrub	Leaves	Chewed	[57]
	*Kleinia squarrosa* Cufod.	Luko (Or)	Shrub	Stem	Brushing	[55]
	*Laggera intermedia* C. B. Clarke	Gimmie (Am)	Herb	Leaves	Crushed	[27]
	*Parthenium hysterophorus* L.	Kalignoole (So)	Herb	Roots	Chewed	[58]
	*Vernonia amygdalina* Del.	Girawa (Am)	Shrub	Leaves	Chewed	[51]
		Eebicha (Or)			Chewed	[59]
	*Vernonia auriculifera* Hiern	Garsach (Me)	Shrub	Roots	Chewed	[60]

Aquifoliaceae	*Ilex mitis* (L.) Radlk.	Mi'esa (Or)	Tree	Twigs		[61]

Balanitaceae	*Balanites aegyptiaca* (L.) Del.	Badana (Or)	Tree	Barks	Chewed	[45]
		Jemo (Am)	Shrub	Roots	Pounded	[62]

Bignoniaceae	*Stereospermum kunthianum* Cham.	Botoroo (Or)	Tree	Stem	Chewed	[47]

Boraginaceae	*Cordia africana* Lam.	Wadesa (Or)	Tree	Barks	Chewed	[45]
		Wanza (Am)		Barks	Powdering	[63]
	*Cynoglossum coeruleum* Hochst. ex A. DC.	Shimgigit (Am)	Herb	Leaves	Holding	[64]
	*Ehretia cymosa* Thonn.	Ulaagaa (Or)	Shrub	Leaves	Chewed	[48]
		Migure (Af)	Tree	Leaves	Powdering	[50]
		Game (Am)				[62]
		Checho (Am)		Leaves	Holding	[65]

Brassicaceae	*Lepidium sativum* L.	Shinfa (Or)	Herb	Seeds	Chewed	[66]

Burseraceae	*Commiphora hodai* Sprague	Hodai (So)	Herb	Roots	Inhaling	[67]

Capparaceae	*Boscia salicifolia* Oliv.	Awo (Ti)	Tree	Leaves	Chewed	[41]
	*Capparis tomentosa* Lam.	Hragama (Or)	Climber	Leave	Chewed	[30]
				Roots	Chewed	[40]
				Barks	Crushed	[49]
		Goraa (Or)		Barks	Chewed	[68]
				Leaves	Heated	[59]
	*Capparis fascicularis* DC.	Hida sare (Or)	Climber	Roots	Chewed	[69]
		Hargama (Or)	Shrub	Roots	Chewed	[40]

Capparidaceae	*Cadaba rotundifolia* Forssk.		Tree	Leaves	Chewed	[70]
	*Crateva adansonii* DC.	Qollaadii (Or)	Shrub	Leaves	Heating	[59]

Caryophyllaceae	*Drymaria cordata* (L.) Schultes	Hakeato (Ke)	Epiphyte	Leaves		[43]

Chenopodiaceae	*Chenopodium opulifolium* Koch	Sinin (Am)	Herb	Leaves	Drink	[50]

Clusiaceae	*Clusia lanceolata* Cambess.	Ulee foonii (Or)	Tree	Leaves		[71]
	*Garcinia livingstonei* T. Anderson	Abuqurto (Or)	Shrub	Stem	Chewed	[72]

Colchicaceae	*Gloriosa superba* L.	Harmel (Or)	Shrub	Leaves	Crushed	[55]

Crassulaceae	*Kalanchoe laciniata* (L.) DC	Endawula (Am)	Herb	Roots	Chewed	[50]
				Roots	Chewed	[57]

Cupressaceae	*Cupressus lusitanica* Mill.	Yeferenj tid (Am)	Tree	Leaves	Decoction	[46]

Cupressaceae	*Juniperus procera* Hochst. ex Endl.	Gaattiraa (Or)	Tree	Bark	Holding	[30]

Cucurbitaceae	*Cucumis ficifolius* A. Rich.	Muchele (Ti)	Herb	Roots	Chewed	[41]
		Yembuay (Am)		Seeds	Crushed	[45]
		Facaa (Or)		Roots	Chewed	[49]
	*Momordica foetida* Schumach.	Yamora misa (Am)	Climber	Leaves	Chewed	[27]
				Roots	Chewed	[50]
		Umbrao (Ke)		Roots		[43]

Ebenaceae	*Euclea divinorum* Hiern	Gunna (Ha)	Shrub	Roots	Drink	[73]
	*Euclea racemosa* L.		Shrub	Roots	Chewed	[29]
		Keleaw (Ti)		Roots	Chewed	[41]
				Roots	Chewed	[42]
		Kliaw (Am)		Roots	Holding	[46]

Euphorbiaceae	*Clutia abyssinica* Jaub. & Spach	Ule foni (Or)	Shrub	Leaves	Holding	[30]
				Leaves	Holding	[48]
		Binjile (Si)	Herb	Roots	Chewed	[74]
	*Phyllanthus sepialis* Mull. Arg	Suamlfer (Or)		Roots		[75]
	*Ricinus communis* L.	Guloo (Am)	Shrub	Roots	Chewed	[50]
				Roots	Chewed	[76]

Fabaceae	*Acacia nilotica* (L.) Willd. ex Del.	Serkema (Or)	Tree	Stem	Decoction	[69]
		Kesel-e (Af)		Leaves	Chewed	[77]
	*Acacia oerfota* (Forssk.) Schweinf.	Garmoyta (Af)	Shrub	Barks	Chewed	[33]
		Ajo (Or)		Twigs	Chewed	[40]
	*Albizia gummifera* (J. F. Gmel.) C.A. Sm.	Muka arbaa (Or)	Tree	Leaves	Rubbed	[48]
	*Calpurnia aurea* (Ait.) Benth.	Digita (Am)	Shrub	Roots	Tied	[52]
		Cadhiw (Ko)		Roots		[78]
	*Colutea abyssinica* Kunth & Bouche	Taetaeta (Ti)	Shrub	Roots	Chewed	[41]
				Stem	Heating	[53]
	*Entada abyssinica* A. Rich.	Galchacha (Si)	Shrub	Barks		[79]
	*Erythrina brucei* Schweinf.	Waleenaa (Or)	Tree	Barks	Chewed	[68]
	*Indigofera spicata* Forssk.	Gimay (Me)	Herb	Roots	Chewed	[60]
	*Millettia ferruginea* (Hochst.) Baker	Dhoqonuu (Or)	Tree	Barks	Chewed	[80]
		Yago (Ke)		Seeds		[43]

Flacourtiaceae	*Dovyalis abyssinica* (A. Rich.) Warb.	Koshim (Am)	Tree	Seeds	Rubbing	[81]
				Seeds	Chewed	[66]

Geraniaceae	*Geranium* sp.	Bedinecho (Da)	Herb	Leaves	Rubbing	[82]
	*Monsonia parvifolia* Schinz			Leaves	Heated	[56]

Lamiaceae	*Clerodendrum myricoides* (Hochst.) R. Br. ex Vatke	Misrich (Am)	Herb	Roots	Crushed	[45]
				Seeds	Chewed	[83]
		Misrach (Or)		Roots	Chewed	[84]
	*Isodon ramosissimus* (Hook.f.) Codd	Dingermiko (Ke)	Herb	Leaves		[43]
	*Mentha pulegium* L.	Setisemhal (Ti)	Herb	Leaves	Chewed	[29]
	*Ocimum urticifolium* Roth	Eyafa (Sk)	Herb	Leaves		[85]
	*Thymus schimperi* Ronniger	Tesne (Ti)	Herb	Whole	Chewed	[41]

Loranthaceae	*Plicosepalus robustus* Wiens & Polhill		Shrub	Leaves	Pounded	[38]
	*Tapinanthus globiferus* (A. Rich.) Tiegh.		Shrub	Leaves	Rubbing	[38]

Malvaceae	*Pavonia urens* Cav.	Maxxannee (Or)	Herb	Roots	Decoction	[86]
	*Sida tenuicarpa* Vollesen	Chfrig (Am)	Shrub	Roots	Brushing	[46]

Meliaceae	*Azadirachta indica* A. Juss.	Talaal (So)	Tree	Leaves	Chewed	[67]
	*Melia azedarach* L.	Niimii (Or)	Tree	Stem	Chewed	[30]
				Stem	Brushing	[45]
		Niim (Am)		Leaves	Chewed	[70]
		Neem (Ti)		Barks	Holding	[87]
		Geed kinin (So)		Leaves	Holding	[58]

Menispermaceae	*Stephania abyssinica* (Quart. Dill. & A. Rich.) Walp.	Shinet (Am)	Climber	Roots	Brushing	[88]

Moraceae	*Ficus palmata* Forssk.	Beles (Am)	Tree	Roots	Chewed	[51]
	*Ficus sur* Forssk.	Shola (Am)	Tree	Barks	Holding	[46]

Myrtaceae	*Eucalyptus* sp.	Baxarsaf (So)	Tree	Roots	Rubbing	[58]

Olacaceae	*Ximenia americana* L	Hudhaa (Or)	Tree	Barks	Powdered	[83]

Oleaceae	*Jasminum abyssinicum* Hochst. ex DC.	Habtselim (Am)	Shrub	Roots	Chewed	[46]
	*Jasminum grandiflorum* L.	Qamaxe (Or)	Tree	Stem	Chewed	[28]
		Bilu (Or)	Shrub	Leaves	Crushed	[55]
	*Olea europaea* L.	Woira (Am)	Tree	Stem	Brushing	[27]
						[52]
				Stem	Chewed	[41]
		Awlie (Ti)		Leaves	Chewed	[42]
				Stem	Heated	[53]
		Wa'era (Ha)		Leaves	Chewed	[73]
				Leaves	Chewed	[45]
		Ejersa (Or)		Leaves	Decoration	[86]
		Ejerssa (Si)		Stem	Chewed	[74]

Oliniaceae	*Olinia rochetiana* A. Juss.	Dalecho (Or)	Tree	Leaves	Holding	[28]
		Chife (Am)		Leaves	Chewed	[81]
		Nolee (Si)		Barks		[79]
				Leaves	Chewed	[59]

Opiliaceae	*Ziziphus mauritiana* Lam.	Kasil (So)	Shrub	Stem	Boiled	[57]

Orobanchaceae	*Orobanche ramosa* L.	Yemako (Si)	Herb	Roots	Chewed	[74]

Oxalidaceae	*Oxalis corniculata* L.	Kakeato (Ke)	Herb	Leaves		[43]
	*Oxalis radicosa* A. Rich.	Solcarindo (Ma)	Herb	Stem	Chewed	[44]

Phytolaccaceae	*Phytolacca dodecandra* L'Her.	Endod (Am)	Shrub	Stem	Chewed	[51]

Polygalaceae	*Securidaca longepedunculata* Fresen.	Etsemena (Am)	Tree	Leaves	Chewed	[47]

Polygonaceae	*Rumex abyssinicus* Jacq.	Mequmeqo (Ti)	Herb	Roots	Crushed	[41]
				Roots	Chewed	[70]
	*Rumex nepalensis* Spreng.	Dhangaggo (Or)	Herb	Roots		[89]

Polypodiaceae	*Drynaria volkensii* Hieron.	Afarfattuu (Or)	Epiphyte	Roots	Holding	[48]
		Kokosso (Si)		Rhizome	Chewed	[90]

Proteaceae	*Faurea speciosa* Welw.	Gero (Ma)	Herb	Roots	Chewed	[44]

Ranunculaceae	*Clematis longicauda* Steud. ex A. Rich.	Zina charo (Sk)	Climber	Leaves		[85]
	*Clematis simensis* Fresen.	Hida Fiti (Or)	Climber	Barks	Crushed	[49]
		Fide (Si)		Stem	Chewed	[91]
	*Ranunculus multifidus* Forssk.	Sherit (Me)	Herb	Roots	Chewed	[60]
		Hogiyo (Ke)		Roots		[43]
	*Thalictrum rhynchocarpum* Dill. & A. Rich	Shunawedi (Ke)	Herb	Roots		[43]

Rosaceae	*Prunus africana* (Hook.f.) Kalkman	Arara (Ha)	Tree		Chewed	[92]
		Omo (Be)		Barks		[89]
	*Prunus persica* (L.) Batsch	Koki (Or)	Tree	Barks	Holding	[47]

Rubiaceae	*Galium boreoaethiopicum* Puff	Mendefgi (Ti)	Herb	Roots	Chewed	[41]
				Roots	Chewed	[42]
	*Gardenia ternifolia* Schumach. & Thonn.	Gambilo (Am)		Shoot	Chewed	[93]
	*Pavetta gardeniifolia* Hochst. ex A. Rich	Qadiidaa (Or)	Shrub	Roots	Pounded	[68]
	*Pentas lanceolata* (Forssk.) Deflers	Afi deshe (Ar)	Herb	Roots	Chewed	[44]

Rutaceae	*Clausena anisata* (Willd.) Hook.f. ex Benth.	Uluma (Or)	Shrub	Roots	Chewed	[28]
		Limich (Am)		Stem Root	Brush	[80]
				Stem	Brush	[65]
		Embricho (Ke)		Leaves		[71]
						[43]
	*Clausena anisata* (Willd.) Hook.f. ex Benth.	Uluma (Or)	Shrub	Roots	Chewed	[28]
				Stem	Brush	[80]
		Limich (Am)		Root		[65]
				Stem	Brush	[71]
		Embricho (Ke)		Leaves		[43]
	*Ruta chalepensis* L.	Cilaadama (Or)	Herb	Leaves	Chewed	[94]
				Leaves	Chewed	[95]
	*Vepris dainellii* (Pichi-Serm.) Kokwaro	Mengereto (Ke)	Tree	Barks		[43]
	*Zanthoxylum chalybeum* Engl.	Ga'ada (Or)	Shrub	Barks	Holding	[45]

Salvadoraceae	*Salvadora persica* L.		Tree	Stem	Brushing	[33]

Sapindaceae	*Dodonaea angustifolia* L. f.	Itacha (Or)	Shrub	Roots	Brushing	[96]

Scrophulariaceae	*Verbascum sinaiticum* Benth.	Timake (Ti)	Shrub	Roots	Chewed	[41]

Simaroubaceae	*Brucea antidysenterica* J.F.Mill.	Qomonyo (Or)	Shrub	Roots	Chewed	[80]
			Tree	Bark		[97]

Solanaceae	*Datura stramonium* L.		Herb	Fruits	Inhaling	[29]
		Manjii (Or)		Seeds	Decoction	[30]
		Asangira (Or)		Seeds	Inhaling	[48]
		Atsefaris (Ma)		Bud	Chewed	[44]
		Astenagir (Am)		Seeds	Inhaling	[50]
		Hitsawats (Ti)		Seeds	Inhaling	[41]
				Seeds	Inhaling	[53]
				Seeds	Powdering	[45]
				Seeds	Chewed	[80]
				Seeds	Decoction	[49]
				Leaves	Grounded	[83]
				Roots	Chewed	[60]
				Leaves	Inhaling	[76]
				Leaves	Decoction	[55]
		Bolute rosun (Me)		Seeds	Inhaling	[98]
		Qamaxari (Or)		Stem	Squished	[54]
	*Nicotiana tabacum* L.	Tamboo (Or)	Herb	Leaves	Chewed	[68]
	*Solanum hastifollium* Hochst.	Alalmo kalbi (Ti)	Shrub	Roots	Chewed	[41]
	*Solanum incanum* L.	Hiddi (Or)	Shrub	Roots	Chewed	[30]
		Xanbax (So)		Fruits	Chewed	[67]
				Roots	Chewed	[49]
				Fruits	Dropping	[68]
	*Solanum marginatum* L. f.	Embuay (Am)	Shrub	Fruits	Dropping	[27]
		Hiddii (Or)			Dropping	[47]

Tiliaceae	*Grewia bicolor* Juss.	Deka (Or)	Shrub	Stem	Brushing	[55]
	*Grewia ferruginea* Hochst. ex A. Rich.	Tsinquayt (Ti)	Tree	Roots	Crushed	[99]

Verbenaceae	*Premna schimperi* Engl.	Dabase (Or)	Shrub	Chewed		[49]
		Xaxesa (Or)		Seeds	Chewed	[83]
		Chcho (Am)	Tree	Roots	Chewed	[46]
				Leaves	Chewed	[98]
				Leaves	Chewed	[100]
	*Premna oligotricha* Baker	Sasa (Ti)	Shrub	Leaves	Chewed	[41]
				Leaves	Chewed	[42]
	*Premna resinos*a (Hochst.) Schauer	Urgessaa (Or)	Tree	Roots	Chewed	[59]

Vitaceae	*Cissus quadrangularis* L.	Gaale-abdi (Or)	Climber	Roots	Chewed	[40]
	*Cyphostemma junceum* (Barker) Desc. ex Wild. R. B. Drumm.	Etse zewye (Ti)	Herb	Whole	Chewed	[41]

Zingiberaceae	*Aframomum corrorima* (Braun) Jansen	Ofiyo (Ke)	Herb	Seeds		[43]
	*Zingiber officinale* Roscoe	Zingibel (Ti)	Herb	Rhizome	Chewed	[29]
				Rhizome	Holding	[101]

Or: Afaan Oromo; So: Somali; Ku: Kunama; Ko: Konta; Ti: Tigre; Am: Amharic; Ha: Hadiya; Ma: Maale; Me: Meinit; Sh: Shinasha; Br: Bertha; Be: Bench; Da: Dawaro; G: Gumuz; Si: Sidama; Sk: Shekkicho; Ari: Ar; Af: Afar; Ke: Keficho.

**Table 2:** Taxonomic diversity of medicinal plants used for toothache treatment.

Family	Number of genera	Percentage	Number of species	Percentage
Asteraceae	10	8.5	12	9.2
Fabaceae	8	6.8	9	6.9
Solanaceae	3	2.5	5	3.8
Euphorbiaceae	4	3.4	4	3.1
Lamiaceae	5	4.3	5	3.8
Oleaceae	3	2.6	4	3.1
Rubiaceae	5	4.3	4	3.1
Acanthaceae	3	2.3	3	2.3
Boraginaceae	3	2.3	3	2.3
Capparidaceae	4	3.4	4	3.1
Ranunculaceae	3	2.3	4	3.1
Rutaceae	4	3.4	4	3.1
Malvaceae	3	2.3	3	2.3
Other 49 families	59	50.4	66	50.7
Total	117	100.0	130	100.0
